# Aberrant Expressional Profiling of Small RNA by Cold Atmospheric Plasma Treatment in Human Chronic Myeloid Leukemia Cells

**DOI:** 10.3389/fgene.2021.809658

**Published:** 2022-02-03

**Authors:** Bo Guo, Wen Li, Yijie Liu, Dehui Xu, Zhijie Liu, Chen Huang

**Affiliations:** ^1^ Department of Cell Biology and Genetics/Key Laboratory of Environment and Genes Related to Diseases, School of Basic Medical Sciences, Xi’an Jiaotong University Health Science Center, Xi’an, China; ^2^ Institute of Genetics and Developmental Biology, Translational Medicine Institute, School of Basic Medical Sciences, Xi’an Jiaotong University Health Science Center, Xi’an, China; ^3^ State Key Laboratory of Electrical Insulation and Power Equipment, Centre for Plasma Biomedicine, Xi’an Jiaotong University, Xi’an, China; ^4^ Key Laboratory of Shaanxi Province for Craniofacial Precision Medicine Research, Xi’an, China

**Keywords:** small RNA, microRNA, expressional profile, cold atmospheric plasma, high-throughput sequencing

## Abstract

Small RNAs (sRNAs), particularly microRNAs (miRNAs), are functional molecules that modulate mRNA transcripts and have been implicated in the etiology of various types of cancer. Cold atmospheric plasma (CAP) is a physical technology widely used in the field of cancer treatment after exhibiting extensive lethality on cancer cells. However, few studies have reported the exact role of miRNAs in CAP-induced anti-cancer effects. The aim of the present study was to determine whether miRNAs are involved in CAP-induced cytotoxicity by using high-throughput sequencing. Our research demonstrated that 28 miRNAs were significantly changed (17 upregulated and 11downregulated) following 24 h of treatment with a room-temperature argon plasma jet for 90 s compared with that of the untreated group in human chronic myeloid leukemia K562 cells. GO enrichment analysis revealed that these target genes were related to cell organelles, protein binding, and single-organism processes. Furthermore, KEGG pathway analysis demonstrated that the target genes of differentially expressed miRNAs were primarily involved in the cAMP signaling pathway, AMPK signaling pathway, and phosphatidylinositol signaling system**.** Taken together, our study demonstrated that CAP treatment could significantly alter the small RNA expression profile of chronic myeloid leukemia cells and provide a novel theoretical insight for elucidating the molecular mechanisms in CAP biomedicine application.

## Introduction

Noncoding RNA (ncRNA) molecules have been extensively explored in the past decades, expanding our understanding of functional elements in the genome (Parts et al., 2012). These noncoding RNAs are then subjected to several different types of small RNAs (sRNAs), a class of 21–24 nucleotides (nt) RNA that play important regulatory roles in the majority of eukaryotes (Lu et al., 2005). Among these molecules, microRNAs (miRNAs) were the first animal small RNA genes to be discovered (Lee et al., 1993), and thousands of examples have been identified in humans (Kozomara and Griffiths-Jones, 2011). The mature miRNA strand is loaded into an argonaute protein-containing complex and guided to target, while the other strand is assumed to be degraded. miRNAs conventionally silence genes by targeting mRNA transcripts via base pair complementarity in the 3′ untranslated region (3′-UTR) (Fang and Rajewsky, 2011). This targeting can induce transcript cleavage, degradation, destabilization, or repression of translation, thus modulating protein levels. Previous studies have shown that small RNA expression can be viewed as a quantitative genetic trait with a downstream influence on gene expression and other phenotypes. It is especially interesting to analyze small RNA transcript levels as intermediate traits potentially causative of downstream effects, as miRNAs have already been implicated in a wide range of human physiological and pathological processes (Lee et al., 2011; Bhattacharya et al., 2018).

Cold atmospheric plasma (CAP), the fourth state of matter other than solid, liquid, and gas, is produced under atmospheric pressure at room temperature with inert gases or air, and is primarily composed of electrons, ions, atoms, molecules, and active radicals (Kong et al., 2009; Bardos and Barankova, 2010). CAP is currently widely used in multiple biomedical fields such as wound healing (Isbary et al., 2010), cell transfection (Xu et al., 2016), endoscope disinfection (Bhatt et al., 2019), dermatology treatment (Bernhardt et al., 2019), anti-virus application (Guo et al., 2018), and especially cancer treatment (Yan et al., 2017). Moreover, CAP treatment can efficiently eliminate tumor cells *in vitro* among various cancers, including lung cancer (Li et al., 2019), melanoma (Alimohammadi et al., 2020), glioblastoma (Kaushik et al., 2020), and bladder cancer (Zhang et al., 2021), thus presenting a tremendous therapeutic potential (Keidar et al., 2011). To date, the most possible mechanism of this anti-tumor capacity is increasing reactive oxygen species (ROS), such as hydrogen peroxide (H_2_O_2_) and superoxide (O_2_^−^), and reactive nitrogen species (RNS), such as nitric oxide (NO) and nitrate (NO_3_^−^), both intracellularly and extracellularly after CAP treatment (Yan et al., 2017). Among these diverse species, H_2_O_2_ has been proved to be the main anti-cancer reactive species causing the death of cancer cells (Bekeschus et al., 2014), which are able to induce DNA and mitochondrial damages that activate cell cycle checkpoints and initiate signaling cascades leading to cell death (Shaw et al., 2021). RNS, such as NO, also plays a non-negligible role since synergistically using H_2_O_2_/NO_2_ in solution can generate an anti-cancer effect more close to that generated by plasma than just using H_2_O_2_ alone (Girard et al., 2016). Besides, it is noteworthy that plasma-generated species also leads to a broad-spectrum activation or inhibition of multiple genes of several signaling pathways, such as p38 mitogen-activated protein kinase (MAPK) (Xia et al., 2019) and phosphoinositide 3-kinase (Eggers et al., 2021), and mitochondrial pathways, such as JNK/cytochrome and c/caspase-9/caspase-3 (Yang et al., 2020a). However, the precise mechanisms underlying CAP treatment-induced changes in gene expression at the transcriptional or post-transcriptional levels require investigation. As described previously, small RNA, especially miRNAs, have the ability to regulate gene expression in a “one-to-multiple” or “multiple-to-one” manner. However, few studies have reported the effect of CAP treatment on small RNA profile alterations in cancers.

The purpose of this study was to investigate the alterations of the small RNA profile following CAP treatment in chronic myeloid leukemia (CML) cells. As a test example with a relatively clean genetic background, we consider human CML, a malignancy defined by a unique molecular event, the BCR-ABL1 oncogene (Minciacchi et al., 2021). Here, we applied an argon (Ar) plasma and explored the small RNA profile using high-throughput sequencing technology. We identified the location and content of the small RNA transcriptome, the extent of sequence and transcript variation, the differential miRNA expression between CAP-treated and non-treated cells, the potential target genes of these differential miRNAs, and the distribution of candidate target genes in the Gene Ontology (GO). This study provides a novel theoretical insight into the potential molecular mechanisms underlying the simultaneous inhibition of signaling pathways induced by CAP treatment in cancer cells.

## Materials and Methods

### Gas Plasma Generation

**FIGURE 1:**
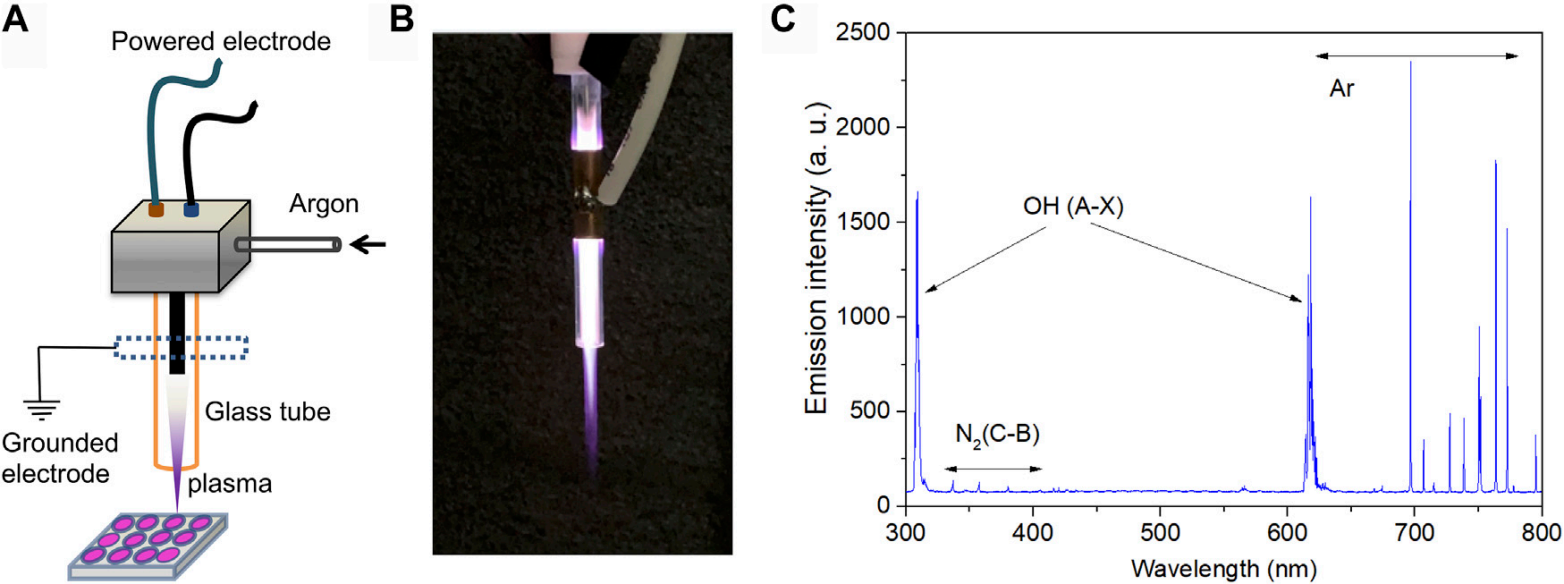
Characteristics of plasma device and the emission profiles. **(A)** Schematic diagram and **(B)** discharge photograph of the argon plasma jet. **(C)** Emission spectra of the CAP jet used in the present study.

An argon plasma jet was used in this study to generate a CAP (Figure 1). The high-voltage electrode was a stainless steel rod with a diameter of 1.61 mm, and was housed in a concentric quartz tube with an inner diameter of 3.91 mm and an outer diameter of 6.34 mm. A copper foil of 20 mm width was wrapped around the tube near the sample-facing end of the high-voltage electrode. The argon plasma was generated at 18 kHz/11 kV with a home-made power supply and argon gas flow of 2 SLM. The dissipated power was obtained by averaging the product of the applied voltage and the plasma current, and was found to be approximately 3.8 W.

### Cell Culture

Human chronic myeloid leukemia K562 cells were preserved at the Biomedical Experimental Center of Xi’an Jiaotong University. Cells were cultured in RPMI-1640 (Biological Industries, Beit Haemek, Israel) supplemented with 10% fetal bovine serum (Biological Industries) and 1% penicillin/streptomycin (Solarbio, Beijing, China) in a humidified incubator (Thermo Scientific, MA, United States) containing 5% CO_2_ at 37°C.

### Cell Viability

Cells were plated into a 12-well plate at a density of 2 × 10^5^/ml for CAP treatment. Cell viability was analyzed using MTT assays (Sigma, MO, United States) at 24, 48, and 72 h after CAP treatment. At the end of the culturing period, 10 μl of MTT solution was added to each well, and the cells were incubated for 4 h at 37°C. Next, the supernatants were discarded and formazan crystals were dissolved in 150 μl of dimethyl sulfoxide. Absorbance was measured at 492 nm using a POLARstar microplate reader (BMG Labtech GmbH, Ortenberg, Germany).

### Measurement of Exocellular Reactive Species

The ROS and RNS content in RPMI-1640 medium were measured with a multi-microplate reader (BMG Labtech GmbH) using a Hydrogen Peroxide Assay Kit (Beyotime, Beijing, China) for H_2_O_2_, Griess Reagent (Beyotime) for nitric oxide (NO), and a Nitrate/Nitrite Assay Kit (Beyotime) for total nitric monoxide according to the manufacturer’s instructions. All measurements were completed within 1 h of argon plasma treatment.

### Sample Collection, RNA Extraction, and Preparation

Cells (1.0 × 10^5^/ml) in 2 ml of medium were seeded into 12-well plates and treated with 90 s of argon plasma, and untreated cells were considered as the control group, and each group had 12 duplicated/samples. After 24 h of incubation, the cells were collected and washed with phosphate-buffered saline three times. Total RNA was extracted using TRIzol Reagent (Invitrogen, CA, United States) according to the manufacturer’s instructions. For RNA quantification and qualification, RNA degradation and contamination were monitored on 1% agarose gels, and RNA purity was checked using a NanoPhotometer spectrophotometer (IMPLEN, CA, United States). RNA concentration was measured using the Qubit RNA Assay Kit in Qubit 2.0 Flurometer (Life Technologies, CA, United States). RNA integrity was assessed using the RNA Nano 6000 Assay Kit of the Agilent Bioanalyzer 2100 system (Agilent Technologies, CA, United States).

### Small RNA Library Construction and Sequencing

Approximately 3 μg of total RNA was used for the small RNA library using NEBNext Multiplex Small RNA Library Prep Set for Illumina (NEB, MA, United States) according to the manufacturer’s instructions, and index codes were added to attribute sequences of each sample. Library quality was assessed on the Agilent Bioanalyzer 2100 system using DNA High Sensitivity Chips. Clustering of the index-coded samples was performed on a cBot Cluster Generation System using TruSeq SR Cluster Kit v3-cBot-HS (Illumina, CA, United States) according to the manufacturer’s instructions. After cluster generation, the library preparations were sequenced on an Illumina Hiseq 2500/2000 platform and 50-bp single-end reads were generated.

### Data Processing and Bioinformatics Analysis

Briefly, clean data (clean reads) were obtained by removing reads containing adapter dimers, junk, and low-quality reads from raw data, and a certain range of length from clean reads was chosen for downstream analyses. The small RNA tags were mapped to search for known miRNAs, and miRbase 20.0 was used as a reference. Custom scripts were used to obtain the miRNA counts as well as base bias on the first position of the identified miRNA with a certain length and on each position of all identified miRNAs, respectively. Novel miRNAs were predicted using two available software (miREvo and mirdeep2) by exploring the secondary structure. miRNA differential expression analysis was performed using the DEGseq (2010) R package. A q value <0.01 and log 2 (fold change) >1 was set as the threshold for significant differential expression by default. To predict the genes targeted by the most abundant miRNAs, computational target prediction algorithms (miRanda 3.3a) were used to identify miRNA binding sites. Finally, GO and Kyoto Encyclopedia of Genes and Genomes (KEGG) enrichment analyses were used on the target gene candidates of differentially expressed miRNAs and to test the high-level functions and utilities of the biological system.

### Quantitative Real-Time PCR

qRT-PCR was performed to verify miRNA expression using the SYBR Green PCR kit according to the manufacturer’s instructions (GenStar, Beijing, China). All reactions were performed in triplicate for each sample using the IQ5 Multicolor Real Time PCR Detection System (Bio-Rad, CA, United States). U6 was used as the control for miRNA and the 2^−ΔΔCt^ method was employed for qRT-PCR analysis. The primer sequences used are listed as follows: miRNA-forward: *5′-ATC​CAG​TGC​GTG​TCG​TG-3′*, miR-10394-5p (RT: *5′-GTC​GTA​TCC​AGT​GCG​TGT​CGT​GGA​GTC​GGC​AAT​TGC​ACT​GGA​TAC​GAC​CAC​AAG​T-3′*, reverse: *5′-TGC​TTC​TGC​AGG​TCC​TGG​TGA-3′*), miR-12136 (RT: *5′-GTC​GTA​TCC​AGT​GCG​TGT​CGT​GGA​GTC​GGC​AAT​TGC​ACT​GGA​TAC​GAC​GGC​CTC​C-3′*, reverse: *5′-TGC​TGA​AAA​AGT​CAT-3′*), miR-1257 (RT: *5′-GTC​GTA​TCC​AGT​GCG​TGT​CGT​GGA​GTC​GGC​AAT​TGC​ACT​GGA​TAC​GAC​GGT​CAG​A-3′*, reverse: *5′-TGC​TAG​TGA​ATG​ATG​GGT-3′*), miR-1291 (RT: *5′-GTC​GTA​TCC​AGT​GCG​TGT​CGT​GGA​GTC​GGC​AAT​TGC​ACT​GGA​TAC​GAC​ACT​GCT​G-3′*, reverse: *5′-TGC​TTG​GCC​CTG​ACT​GAA​GAC-3′*), miR-19a-3p (RT: *5′-GTC​GTA​TCC​AGT​GCG​TGT​CGT​GGA​GTC​GGC​AAT​TGC​ACT​GGA​TAC​GAC​TCA​GTT​T-3′*, reverse: *5′-TGC​TTG​TGC​AAA​TCT​ATG​CA-3′*), miR-320b (RT: *5′-GTC​GTA​TCC​AGT​GCG​TGT​CGT​GGA​GTC​GGC​AAT​TGC​ACT​GGA​TAC​GAC​TTG​CCC​T-3′*, reverse: *5′-TGC​TAA​AAG​CTG​GGT​TGA​G-3′*), miR-619-5p (RT: *5′-GTC​GTA​TCC​AGT​GCG​TGT​CGT​GGA​GTC​GGC​AAT​TGC​ACT​GGA​TAC​GAC​GGC​TCA​T-3′*, reverse: *5′-TGC​TGC​TGG​GAT​TAC​AGG​C-3′*), miR-200a-3p (RT: *5′-GTC​GTA​TCC​AGT​GCG​TGT​CGT​GGA​GTC​GGC​AAT​TGC​ACT​GGA​TAC​GAC​ACA​TCG​T-3′*, reverse: *5′-TGC​TTA​ACA​CTG​TCT​GGT​A-3′*), miR-10b-5p (RT: *5′-GTC​GTA​TCC​AGT​GCG​TGT​CGT​GGA​GTC​GGC​AAT​TGC​ACT​GGA​TAC​GAC​CAC​AAA​T-3′*, reverse: *5′-TGC​TTA​CCC​TGT​AGA​ACC​GA-3′*), miR-30a-5p (RT: *5′-GTC​GTA​TCC​AGT​GCG​TGT​CGT​GGA​GTC​GGC​AAT​TGC​ACT​GGA​TAC​GAC​CTT​CCA​G-3′*, reverse: *5′-TGC​TTG​TAA​ACA​TCC​TCG​A-3′*), and U6 (RT: *5′-CGC​TTC​ACG​AAT​TTG​CGT​GTC​AT-3′*, forward: *5′-GCT​TCG​GCA​GCA​CAT​ATA​CTA​AAA​T-3′*, reverse: *5′-CGC​TTC​ACG​AAT​TTG​CGT​GTC​AT-3′*).

### Statistics

All experiments were performed at least in triplicate unless stated otherwise. Statistical analyses were performed out using GraphPad Prism software. For the cell viability assay, measurement of reactive species, and miRNA expression, Student’s *t*-test was used to analyze the differences between two independent groups. Mean ± SD were reported and *p* <0.05 was considered statistically significant.

## Results

### Plasma Discharge Parameters and Characters

Figure 1A illustrates the argon plasma jet setup used in this study. Figure 1B shows a photograph of the plasma jet and the plasma discharge. The surfaces of the RPMI-1640 medium were 20 and 30 mm from the tube nozzle and the distal end of the high-voltage electrode, respectively. Certain reactive species were observed, such as N_2_ (C-B) at 340–400 nm, OH (A-X) at 310/620 nm, and Ar at 700–800 nm (Figure 1C).

### *In Vitro* Anti-Cancer Effect and Reactive Species Generation of CAP

**FIGURE 2:**
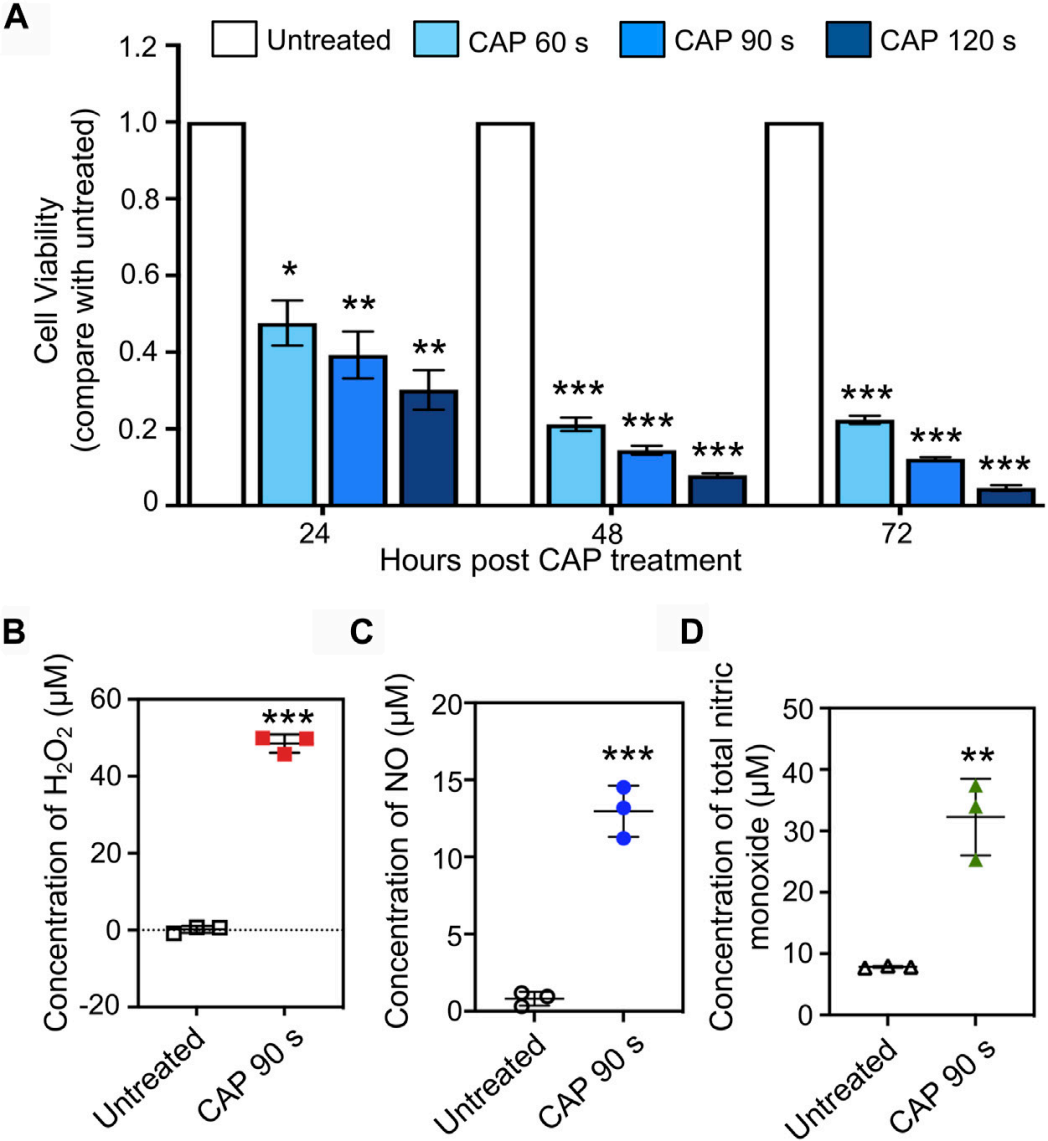
*In vitro* anti-cancer effect and reactive species generation of CAP. **(A)** Cell viability of human chronic myeloid leukemia K562 cells at 24, 48, and 72 h post-CAP treatment in different periods (*n* = 5, *p < 0.05, **p < 0.01, ***p < 0.001). **(B–D)** H_2_O_2_, NO, and total nitric monoxide production in cell culture medium after 90 s of CAP treatment (*n* = 3, **p < 0.01, ***p < 0.001).

Human chronic myeloid leukemia K562 cells were seeded into 12-well plates, and treated with CAP for 60, 90, and 120 s. Cell viability was measured every 24 h after CAP treatment. Compared with that of untreated cells, the decrease in K562 cell viability was reduced to 30–48% at 24 h and 5–22% at 72 h, in a treatment time-dependent manner (Figure 2A, *p < 0.05, **p < 0.01, ***p < 0.001). To prepare enough RNA for small RNA library construction and sequencing, cells treated with CAP for 90 s at 24 h were collected. The reactive species in cell culture medium after CAP treatment were detected; for a 90-s CAP treatment, H_2_O_2_, NO, and total nitric monoxide reached concentrations of 48.4, 13.2, and 32.3 μM, respectively (Figures 2B–D, **p < 0.01, ***p < 0.001).

### Small RNA Sequencing

**FIGURE 3:**
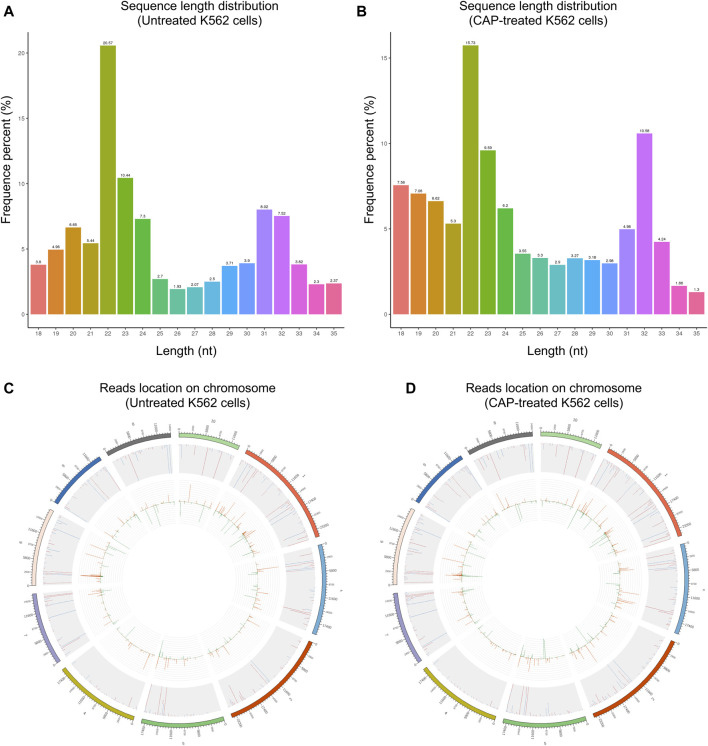
The length distribution of the clean reads sequence and location of clean reads on each chromosome. **(A,C)** Untreated K562 cells. **(B,D)** CAP-treated K562 cells. The top 10 scaffolds are presented here.

Through Solexa high-throughput sequencing, 12,890,967 total reads and 12,567,749 total reads from mock and CAP-treated cell libraries were obtained, respectively. After removing the reads containing poly-N, with 5′ adapter contaminants, without 3′ adapter or the insert tag, containing poly A, T, G, or C and low-quality reads, 11,617,426 (90.1%) and 11,926,789 (94.9%) high-quality clean reads from the two groups were extracted (Table 1). For the downstream analyses, the sequence length distributions of the two libraries were analyzed, and a certain length range was selected. The length of the clean reads peaked at 21–22 nt, which is generally in the range of miRNAs (Figures 3A,B). Approximately 92.73 and 92.88% of sRNA could be mapped to the genome in the two groups using bowtie software (Langmead et al., 2009) (Table 2). In addition, the density statistics of the reads of each chromosome on the genome of each sample were carried out, and the distribution of the reads on each chromosome was checked by Circos mapping (Figures 3C,D). Next, reads of rRNA, tRNA, snoRNA, and other snRNAs were annotated and then removed for the following analysis (Supplementary Figure S1; Supplementary Table S1).

**TABLE 1:** Raw data quality control

Sample	Total reads	N% > 10%	Low quality	5 adapter contamine	3 adapter null or insert null	With poly A/T/G/C	Clean reads
Control	12,890,967	157	107,599	2,210	1,065,569	98,006	11,617,426
CAP	12,567,749	11	49,857	980	530,800	59,312	11,926,789

**TABLE 2:** Reads mapping to the reference sequence

Sample	Total sRNA	Mapped sRNA	“+” Mapped sRNA	“−” Mapped sRNA
Control	9,868,745	9,151,076	5,864,439	3,286,637
CAP	10,107,046	9,387,723	6,416,595	2,971,128

### Differentially Expressed Known miRNAs

**FIGURE 4:**
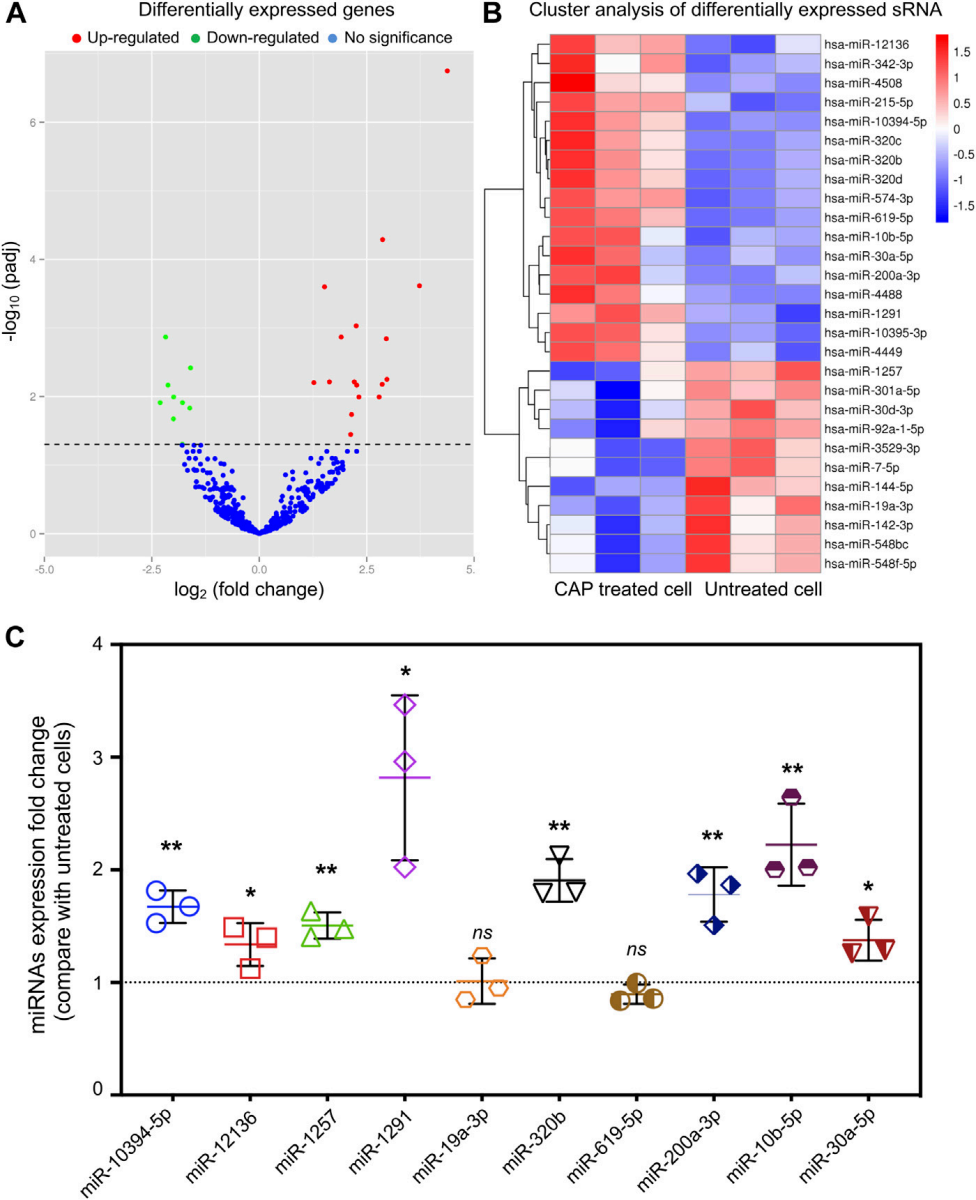
Volcano plot and heat map of differentially expressed miRNAs obtained from the comparison of CAP-treated and untreated K562 cells. **(A)** Each point in the volcano plot represents one miRNA. Red points represent upregulated ones, green points represent downregulated ones, and blue points represent no significant changes of miRNAs. **(B)** The abscissa represents different groups, the ordinate represents the different miRNAs compared in the groups, and the color blocks at different positions represent the relative miRNA expressions. **(C)** miRNA expression of fold change detected by qRT-PCR in K562 cells after 90 s of CAP treatment (*n* = 3, *p < 0.05, **p < 0.01, *ns* = no significance).

Mapped small RNA tags and miRBase 20.0 were used to identify known miRNAs. A total of 1,804 known mature miRNAs and 1,682 miRNA precursors were obtained (Table 3). miRNA expression levels were estimated by transcript per million (TPM), and differential expression analysis was performed between the untreated and CAP-treated groups (Supplementary Figure S2; Supplementary Tables S2, S3). Compared with that of untreated cells, 17 miRNAs (hsa-miR-10395-3p, hsa-miR-320d, hsa-miR-4488, hsa-miR-574-3p, hsa-miR-320c, hsa-miR-215-5p, hsa-miR-12136, hsa-miR-4449, hsa-miR-320b, hsa-miR-619-5p, hsa-miR-342-3p, hsa-miR-10394-5p, hsa-miR-10b-5p, hsa-miR-200a-3p, hsa-miR-4508, hsa-miR-30a-5p, hsa-miR-1291) were significantly upregulated, while 11 miRNAs (hsa-miR-19a-3p, hsa-miR-3529-3p, hsa-miR-7-5p, hsa-miR-144-5p, hsa-miR-142-3p, hsa-miR-1257, hsa-miR-548bc, hsa-miR-548f-5p, hsa-miR-30d-3p, hsa-miR-301a-5p, hsa-miR-92a-1-5p) were downregulated in CAP-treated cells (Figures 4A,B, *p* < 0.05, fold change >1). For validation, we assessed the expression of 10 randomly selected miRNAs in K562 cells after 90 s of CAP treatment using qRT-PCR. Notably, in response to CAP treatment, seven of these miRNAs (miR-10394-5p, miR-12136, miR-1291, miR-302b, miR-200a-3p, miR-10b-5p, and miR-30a-5p) were increased at 24 h post-treatment, whereas levels of miR-19a-3p and miR-619-5p showed no significant difference, exhibiting a similar trend to that of RNA sequencing data (Figure 4C, *p <0.05, **p <0.01, ns = no significance).

**TABLE 3:** Known miRNAs obtained

Sample	Mapped mature	Mapped hairpin	Mapped unique sRNA	Mapped total sRNA
Control	901	847	4,284	2,455,651
CAP	903	835	4,452	1,781,898

### GO Analysis of the Candidate Target Genes of Differentially Expressed miRNAs

**FIGURE 5:**
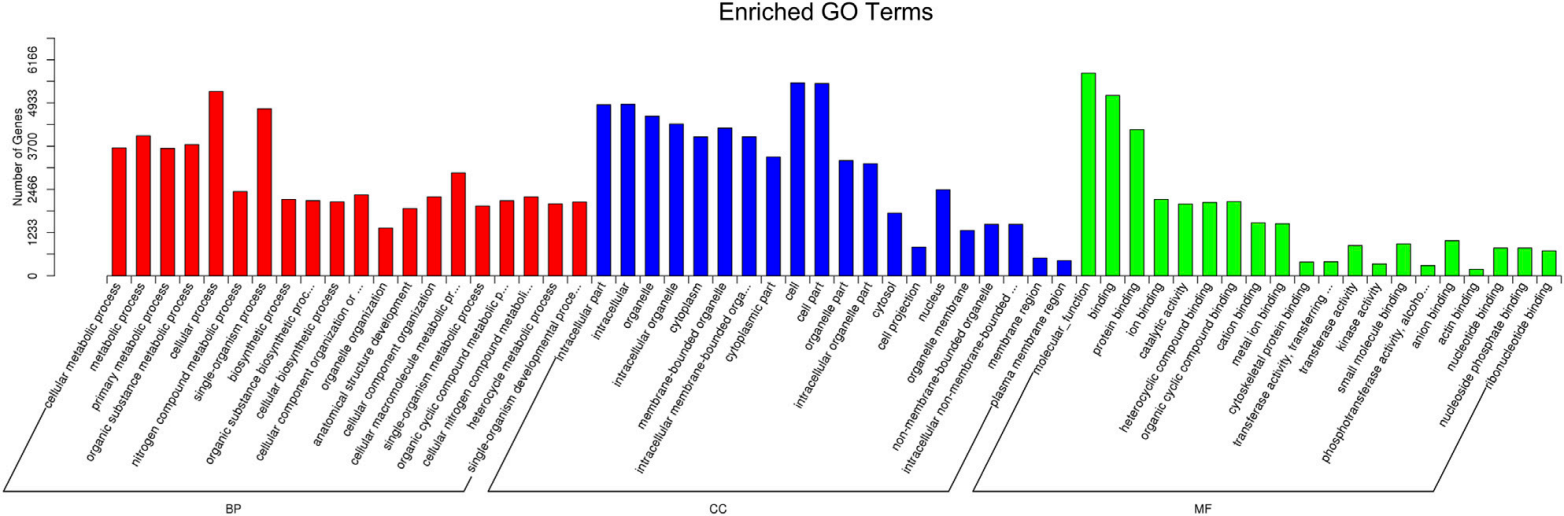
GO function classification target genes of known differential expressed miRNAs in K562 cells after CAP treatment. Abscissa: class classification. The three different classifications represent the three basic classifications of GO terms. From left to right, they are biological process (BP), cellular component (CC), and molecular function (MF).

The target genes of miRNAs were predicted by miRanda and RNAhybrid. The distribution of target gene candidates of differentially expressed miRNAs in the GO was compared with that of the reference group, and the number of genes of the significantly enriched GO terms was counted to determine which biological functions were significantly correlated (Supplementary Table S4). GO enrichment analysis showed that these target genes were related to metabolic process, cellular process, single-organism process, cell, cell part, and protein binding process (Figure 5; Supplementary Figure S3).

### KEGG Pathway Analysis of the Candidate Target Genes of Differentially Expressed miRNAs

**FIGURE 6:**
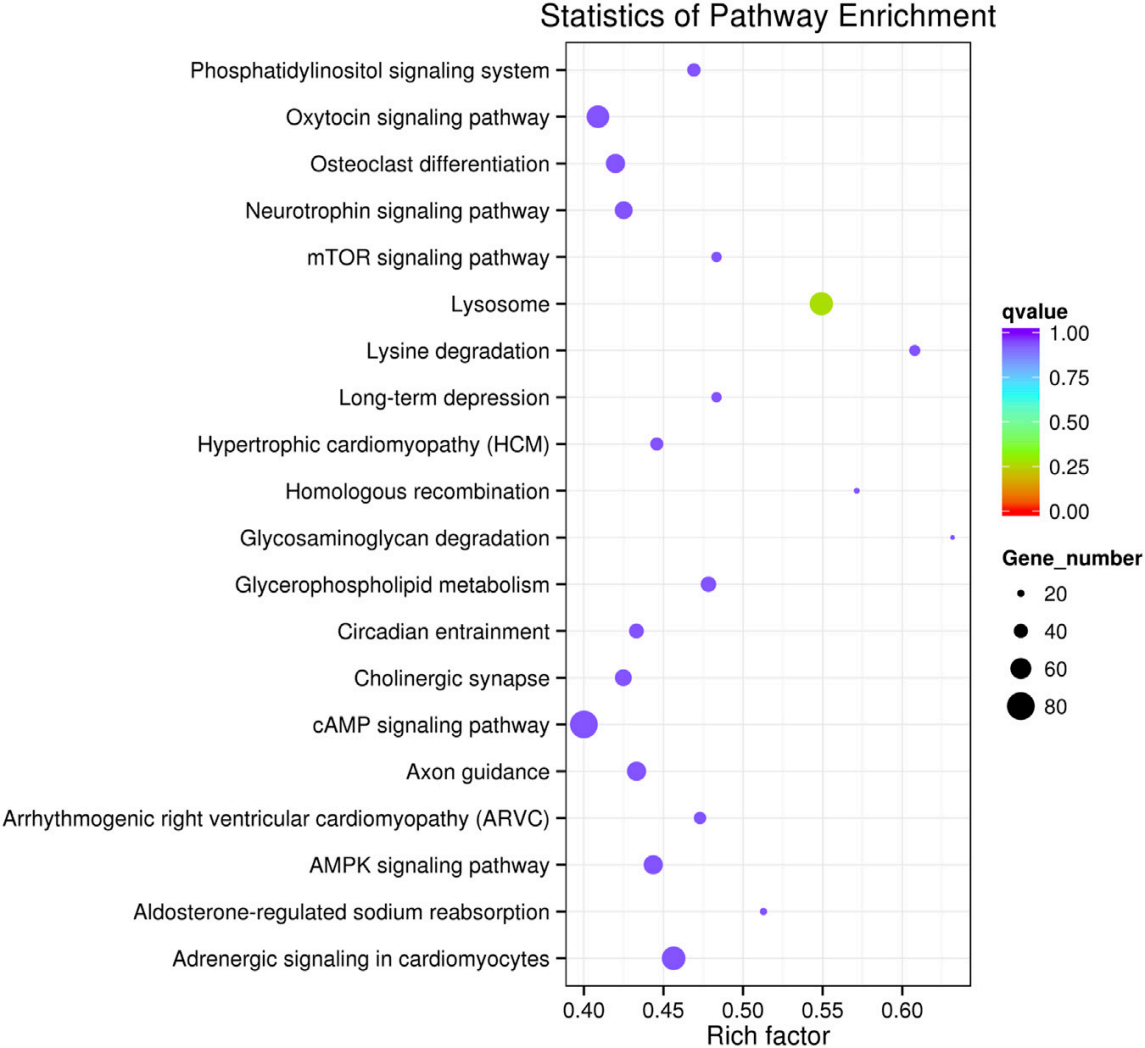
KEGG pathway analysis of the target genes of known differentially expressed miRNAs in K562 cells after CAP treatment. Horizontal axis: rich factor. The larger the point, the higher the enrichment degree, the more candidate target genes in this pathway, and the color of the point corresponds to a different q value range. Vertical axis: the definition of the pathway.

The target genes were mapped to the reference pathways recorded in the KEGG database to identify the biological pathways through which differentially expressed miRNAs were involved in CAP-induced cell death (Supplementary Table S5). KEGG pathway analysis revealed 20 major pathways occupied by the most abundant target genes of differentially expressed miRNAs. In CAP-treated cells, the target gene enrichment pathways included the cAMP, mTOR, and AMPK signaling pathways and the phosphatidylinositol signaling system (Figure 6). These results revealed the potential function of miRNA targets, which may form a regulatory network and play a vital role in cancer progression.

## Discussion

Target therapy failure is the primary reason for increased mortality in cancer-related deaths. Crosstalk among cancer hallmarks such as energy metabolism, growth signaling, immune escape, and redox homeostasis grants plasticity as an important characteristic of cancer cells. Consequently, this plasticity provides neoplastic cells with the capacity to evade inhibition at limited target sites (Chang and Pearce, 2016). For example, glycolysis inhibition may be rescued by activating the HIF pathway (Wellmann et al., 2004) or bypassed by shifting to the oxidative phosphorylation of cancer cells (Fantin et al., 2006). Therefore, a broad-spectrum targeting therapy for cancer hallmarks is highly desirable; however, it currently remains unknown. A novel candidate is ROS and RNS produced with cold atmospheric plasma that are exogenously generated in gas first and then transported to cell-containing solutions to modulate cellular processes (Kong et al., 2009). CAP treatment is conventionally performed at or near room temperature. In comparison with current ROS-generating compounds, a key advantage of CAP is the diversity of its reactive oxygen and nitrogen species (RONS), including H_2_O_2_, singlet oxygen (^1^O_2_), and superoxide (O_2_^•−^), and RNS such as nitric oxide (NO^•^) and peroxynitrite (ONOO^−^) (Kong et al., 2009). There is considerable *in vitro* and *in vivo* evidence of CAP activity in various cancers, and its potential for clinical translation has been demonstrated in, for example, a combination strategy with immunogenic cell death in melanoma cells (Lin et al., 2019). In this study, we developed a study model consisting of room-temperature argon plasma for RONS generation and human chronic myeloid leukemia k562 cells to test whether this CAP jet could achieve a robust killing effect. As a result, longer treatment time drives the k562 cells to undergo a more significant reduction in cell viability than in that of untreated cells, which is consistent with the findings of our previous *in vitro* and *in vivo* studies.

To understand and improve the efficacy of CAP in cancer treatment, it is essential to gain insights into the underlying mechanisms of action. To date, the main casts of CAP related to biological function are believed to be RONS, which easily penetrates the cell membrane and diffuses into the cytosol (Fisher, 2009), leading to the regulation of gene expression among diverse signaling pathways. For example, the MAPK pathway was suppressed by decreasing MMP9 expression by CAP in cervical cancer cells (Li et al., 2016), and p53 or p21 was dysregulated by CAP in prostate cancer cells (Weiss et al., 2015). Currently, the molecular mechanism by which CAP affects gene expression is unclear. miRNAs can regulate gene expression by targeting mRNAs and act as master regulators to modulate various complex biological processes, including cell growth, apoptosis, proliferation, and differentiation. It necessary to determine whether miRNAs play a role in CAP-induced cancer cell lethality (Brany et al., 2021). Yang et al. (2020b) reported that a helium atmospheric-pressure plasma jet treatment induced miR-203a overexpression and then led to the degradation of its target gene BIRC5, thereby suppressing cell proliferation and accelerating cell apoptosis in human lung cancer. Another study identified miR-19a-3p as a mediator of the cell proliferation-inhibitor effect of CAP in MCF-7 breast cancer cells (Lee et al., 2016). In the present study, we explored the small RNA profile using high-throughput sequencing technology and identified 17 upregulated and 11 downregulated miRNAs, and the candidate target genes of these differentially expressed miRNAs in GO. To the best of our knowledge, this is the first study to screen the involvement of miRNAs in the anti-proliferative effect of CAP on cancer cells.

To continue the study, selecting one or more closely associated miRNAs is extremely helpful for exploring the molecular mechanism of the anti-cancer effect of CAP. Based on their role and function in cancer progression, miRNAs can be divided into two families: oncogenic and tumor suppressor. Therefore, the first principle is to select upregulated tumor suppressor miRNAs or downregulated oncogenic miRNAs after CAP treatment. However, it should be noted that even the expression of the same miRNA is not exactly the same among different tumors or tissues. Thus, the tips under the first principle are to consider the genetic background and baseline of miRNA expression in selected cancers. Second, the choice should be based on the consideration of cell phenotype changes and GO or KEGG enrichment analyses after CAP treatment. For example, our present data show AMPK signaling to be a promising pathway (Figure 6); thus, miR-301a and miR-200a (Figure 4) should be chosen for further study, as their targeting relationship has already been verified in lung and breast cancers (Tsouko et al., 2015; Li et al., 2020).

In conclusion, this study verified the anti-tumor effect of CAP in human chronic myeloid leukemia cells and screened differential miRNA expression profiles through high-throughput sRNA sequencing and experimental validation. In addition, GO and KEGG enrichment analysis of the differentially expressed candidate target genes provides a promising option to identify potential miRNAs for further research. Hopefully, our results may contribute to establishing a theoretical perspective for uncovering the molecular mechanisms of CAP biomedicine application. However, this study might have some limitations that merit consideration. First, we did not examine the correlation between the expression of candidate miRNAs and ROS or RNS quantitation. This may help to explain how CAP-produced reactive species affect the expression of small RNA and would be addressed in our future research. Second, we performed the current study on only one type of human cancer. Further research involving more cell lines from multiple cancer types would be a great addition to the present study.

## Data Availability

The datasets presented in this study can be found in online repositories. The names of the repository/repositories and accession number(s) can be found below: https://www.ncbi.nlm.nih.gov/sra PRJNA788344.
