# Supplementary material for: Aberrant Expressional Profiling of Small RNA by Cold Atmospheric Plasma Treatment in Human Chronic Myeloid Leukemia Cells
Source: Front Genet. 2022 Feb 3;12:809658. doi: 10.3389/fgene.2021.809658 (PMC8851033; doi:10.3389/fgene.2021.809658)
Supplement: Supplementary file 1 [file DataSheet1.docx]

Supplementary Information

Aberrant expressional profiling of small RNA by cold atmospheric plasma treatment in human chronic myeloid leukemia cells

Bo Guo^1,2#^, Wen Li^1,2#^, Yijie Liu^1,2^, Dehui Xu^3^, Zhijie Liu^3^, Chen Huang^1,2^*

^1^ Department of Cell Biology and Genetics/Key Laboratory of Environment and Genes Related to Diseases, School of Basic Medical Sciences, Xi’an Jiaotong University Health Science Center, Xi’an, China

^2^ Institute of Genetics and Developmental Biology, Translational Medicine Institute, School of Basic Medical Sciences, Xi’an Jiaotong University Health Science Center, Xi’an, China

^3^ State Key Laboratory of Electrical Insulation and Power Equipment, Centre for Plasma Biomedicine, Xi’an Jiaotong University, Xi’an, China

^#^These authors contributed equally to this work.

*** Correspondence:**Chen Huang
hchen@xjtu.edu.cn

**This PDF file includes:**

Supplementary Table S1 to S5

Figure S1 to S3

**Table S1. small RNA category.**

| Types | Control | Control (percent) | CAP | CAP (percent) |
| --- | --- | --- | --- | --- |
| total | 9151076 | 100.00% | 9387723 | 100.00% |
| known_miRNA | 2455651 | 26.83% | 1781898 | 18.98% |
| rRNA | 66762 | 0.73% | 213047 | 2.27% |
| tRNA | 41339 | 0.45% | 99240 | 1.06% |
| snRNA | 34751 | 0.38% | 37739 | 0.40% |
| snoRNA | 204546 | 2.24% | 185747 | 1.98% |
| repeat | 969377 | 10.59% | 655683 | 6.98% |
| novel_miRNA | 13583 | 0.15% | 5251 | 0.06% |
| exon | 606920 | 6.63% | 1387452 | 14.78% |
| intron | 1189089 | 12.99% | 1780902 | 18.97% |
| other | 3569058 | 39.00% | 3240764 | 34.52% |

**Table S2. Quantification of miRNA expression level by TPM (Parts of data).**

| sRNA | Control.readcount | CAP.readcount | Control.tpm | CAP.tpm |
| --- | --- | --- | --- | --- |
| let-7c-5p | 259 | 191 | 99.8204773037526 | 103.07926363628 |
| let-7e-5p | 2035 | 2110 | 784.30375 | 1138.72904 |
| miR-100-5p | 1071 | 473 | 412.771163 | 255.26959 |
| miR-101-3p | 2992 | 2166 | 1153.13849 | 1168.95123 |
| miR-103b | 7128 | 12294 | 2747.18287 | 6634.85061 |
| miR-106a-5p | 44 | 53 | 16.9579189 | 28.6031465 |
| miR-10b-5p | 204 | 230 | 78.6230786 | 124.126862 |
| miR-122-5p | 554 | 42 | 213.515616 | 22.6666444 |
| miR-122b-3p | 554 | 42 | 213.515616 | 22.6666444 |
| miR-129-5p | 335 | 157 | 129.111428 | 84.7300753 |
| miR-140-5p | 212 | 124 | 81.7063366 | 66.9205691 |
| miR-146b-3p | 948 | 2806 | 365.366071 | 1514.34772 |
| miR-21-3p | 101 | 153 | 38.9261321 | 82.5713473 |
| miR-218-5p | 4349 | 1913 | 1676.13612 | 1032.41168 |
| miR-222-3p | 934 | 1057 | 359.97037 | 570.443883 |
| miR-23a-3p | 1211 | 2118 | 466.728178 | 1143.04649 |
| miR-30e-5p | 3890 | 2161 | 1499.2342 | 1166.25282 |
| miR-3180 | 1002 | 681 | 386.178063 | 367.523448 |
| miR-34a-5p | 3 | 17 | 1.15622174 | 9.17459415 |
| miR-365a-5p | 266 | 237 | 102.518328 | 127.904636 |
| miR-4326 | 320 | 406 | 123.330319 | 219.110895 |
| miR-449c-5p | 20 | 12 | 7.70814497 | 6.4761841 |
| miR-454-3p | 517 | 384 | 199.255547 | 207.237891 |
| miR-505-5p | 12 | 16 | 4.62488698 | 8.63491214 |

**Table S3. Differential expressed miRNA from CAP-treated K562 cells. (Parts of data)**

| sRNA | Control.readcount | CAP.readcount | | Log2Foldchange | P value (adj.) |
| --- | --- | --- | --- | --- | --- |
| let-7c-5p | 110.515182 | | 295.307025 | 1.2058 | 0.17202 |
| miR-100-5p | 335.23949 | | 1605.91196 | 1.7319 | 0.094519 |
| miR-10394-5p | 0.30723351 | | 10.6155502 | 2.8601 | 0.0066286 |
| miR-12136 | 5.63892362 | | 96.3981888 | 2.9557 | 0.0014381 |
| miR-1257 | 204.706051 | | 22.8035203 | -2.3064 | 0.012264 |
| miR-125b-5p | 39.2923275 | | 191.802995 | 1.6726 | 0.12538 |
| miR-1291 | 2.65202958 | | 20.7134798 | 2.1263 | 0.035736 |
| miR-142-3p | 831.44573 | | 163.816374 | -1.9916 | 0.010152 |
| miR-149-5p | 6.43127999 | | 18.6049143 | 1.4435 | 0.07972 |
| miR-19a-3p | 266.56682 | | 47.1166525 | -2.1794 | 0.0013576 |
| miR-20a-5p | 29858.5556 | | 7589.81763 | -1.6323 | 0.07972 |
| miR-21-5p | 139407.413 | | 97122.5321 | -0.4837 | 0.51218 |
| miR-320b | 110.75898 | | 384.283771 | 1.6328 | 0.0061161 |
| miR-4449 | 2.55958126 | | 102.526053 | 2.9698 | 0.005618 |
| miR-503-5p | 11.9866321 | | 28.4470728 | 1.0628 | 0.21088 |
| miR-517a-3p | 8.46698959 | | 44.2487359 | 1.7714 | 0.08056 |
| miR-548bc | 183.669102 | | 44.731812 | -1.7863 | 0.012264 |
| miR-619-5p | 3.40861754 | | 23.7401391 | 2.211 | 0.0061161 |
| miR-651-5p | 121.829657 | | 39.6564441 | -1.3674 | 0.09683 |
| miR-7974 | 557.214358 | | 119.156987 | -1.7937 | 0.05138 |
| miR-92a-1-5p | 886.541476 | | 193.874071 | -1.7899 | 0.04958 |

**Table S4. Go enrichment analysis results. (Parts of data)**

| GO_accession | Description | Term_type | p Value |
| --- | --- | --- | --- |
| GO:0098590 | plasma membrane region | cellular_component | 6.31E-08 |
| GO:0044711 | single-organism biosynthetic process | biological_process | 7.09E-08 |
| GO:0097458 | neuron part | cellular_component | 1.24E-07 |
| GO:0070013 | intracellular organelle lumen | cellular_component | 1.49E-07 |
| GO:0034645 | cellular macromolecule biosynthetic process | biological_process | 1.64E-07 |
| GO:1902589 | single-organism organelle organization | biological_process | 2.53E-07 |
| GO:0044271 | cellular nitrogen compound biosynthetic process | biological_process | 2.57E-07 |
| GO:0048468 | cell development | biological_process | 2.62E-07 |
| GO:0043233 | organelle lumen | cellular_component | 2.95E-07 |
| GO:0044463 | cell projection part | cellular_component | 3.26E-07 |
| GO:0009966 | regulation of signal transduction | biological_process | 3.40E-07 |
| GO:0001883 | purine nucleoside binding | molecular_function | 2.35E-05 |
| GO:0032550 | purine ribonucleoside binding | molecular_function | 2.35E-05 |
| GO:0016310 | phosphorylation | biological_process | 4.32E-07 |
| GO:0012505 | endomembrane system | cellular_component | 4.32E-07 |
| GO:0009059 | macromolecule biosynthetic process | biological_process | 4.99E-07 |
| GO:0022008 | neurogenesis | biological_process | 5.71E-07 |
| GO:0090304 | nucleic acid metabolic process | biological_process | 6.02E-07 |
| GO:0016740 | transferase activity | molecular_function | 6.84E-07 |
| GO:0016773 | phosphotransferase activity, alcohol group as acceptor | molecular_function | 4.80E-06 |
| GO:0006811 | ion transport | biological_process | 1.09E-05 |
| GO:0031981 | nuclear lumen | cellular_component | 1.11E-05 |
| GO:0032879 | regulation of localization | biological_process | 5.12E-06 |

**Table S5. KEGG pathway terms (Top 20)**

| pathway_term | rich_factor | q value | gene_number |
| --- | --- | --- | --- |
| Lysosome | 0.54918033 | 0.27297145 | 67 |
| Lysine degradation | 0.60784314 | 0.93621537 | 31 |
| Adrenergic signaling in cardiomyocytes | 0.45637584 | 0.93621537 | 68 |
| Glycerophospholipid metabolism | 0.47826087 | 0.93621537 | 44 |
| AMPK signaling pathway | 0.44354839 | 0.93621537 | 55 |
| Phosphatidylinositol signaling system | 0.4691358 | 0.93621537 | 38 |
| Arrhythmogenic right ventricular cardiomyopathy (ARVC) | 0.47297297 | 0.93621537 | 35 |
| Homologous recombination | 0.57142857 | 0.93621537 | 16 |
| Glycosaminoglycan degradation | 0.63157895 | 0.93621537 | 12 |
| Axon guidance | 0.43307087 | 0.93621537 | 55 |
| mTOR signaling pathway | 0.48333333 | 0.93621537 | 29 |
| Long-term depression | 0.48333333 | 0.93621537 | 29 |
| Aldosterone-regulated sodium reabsorption | 0.51282051 | 0.93621537 | 20 |
| Hypertrophic cardiomyopathy (HCM) | 0.44578313 | 0.93621537 | 37 |
| Neurotrophin signaling pathway | 0.425 | 0.93621537 | 51 |
| Osteoclast differentiation | 0.41984733 | 0.93621537 | 55 |
| Circadian entrainment | 0.43298969 | 0.93621537 | 42 |
| Cholinergic synapse | 0.42477876 | 0.93621537 | 48 |
| Oxytocin signaling pathway | 0.40880503 | 0.93621537 | 65 |
| cAMP signaling pathway | 0.4 | 0.93621537 | 80 |


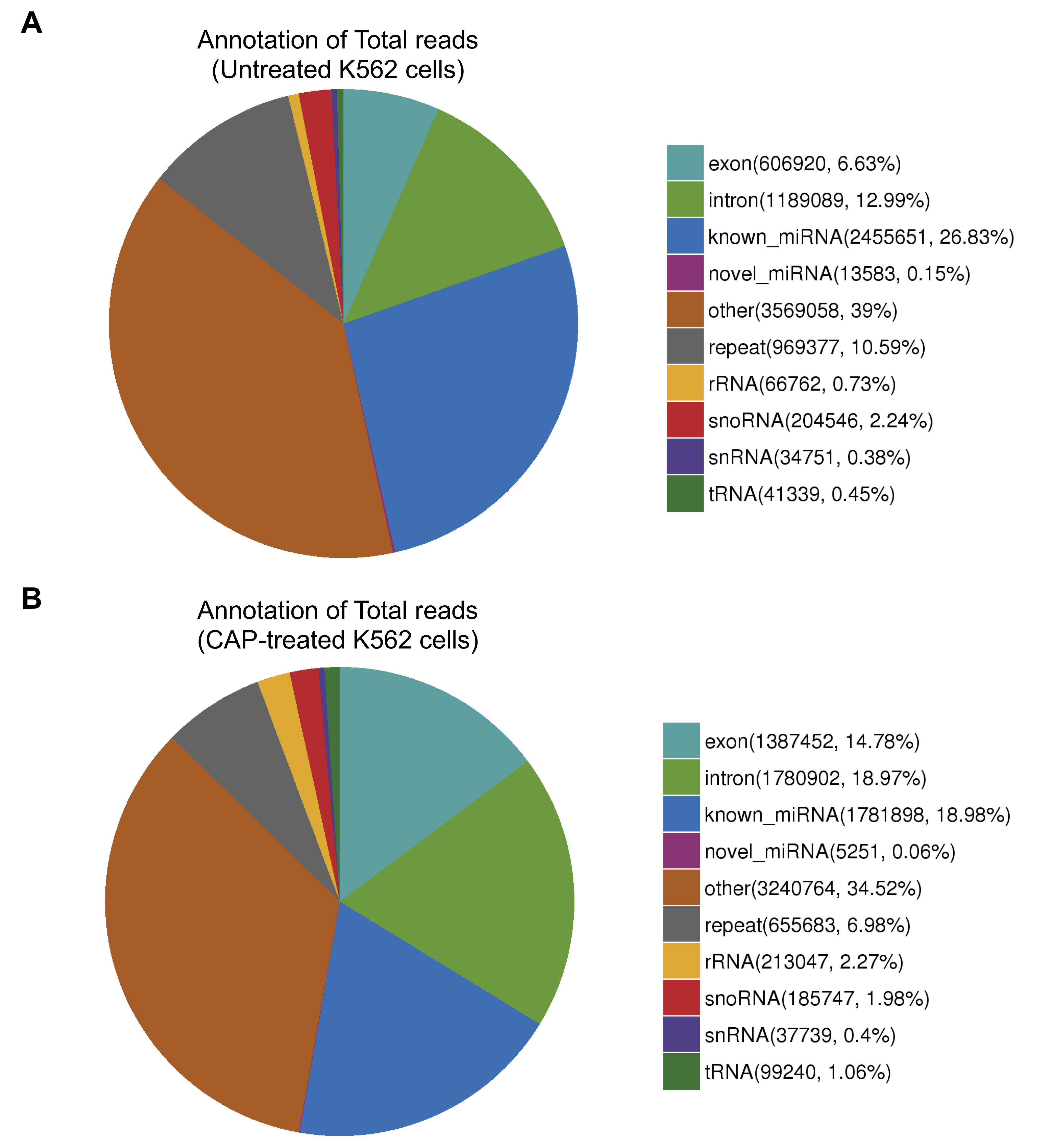


**Figure. S1**. **The sRNA classification annotation statistics of the clean reads.** (A) Untreated K562 cells. (B) CAP-treated K562 cells.


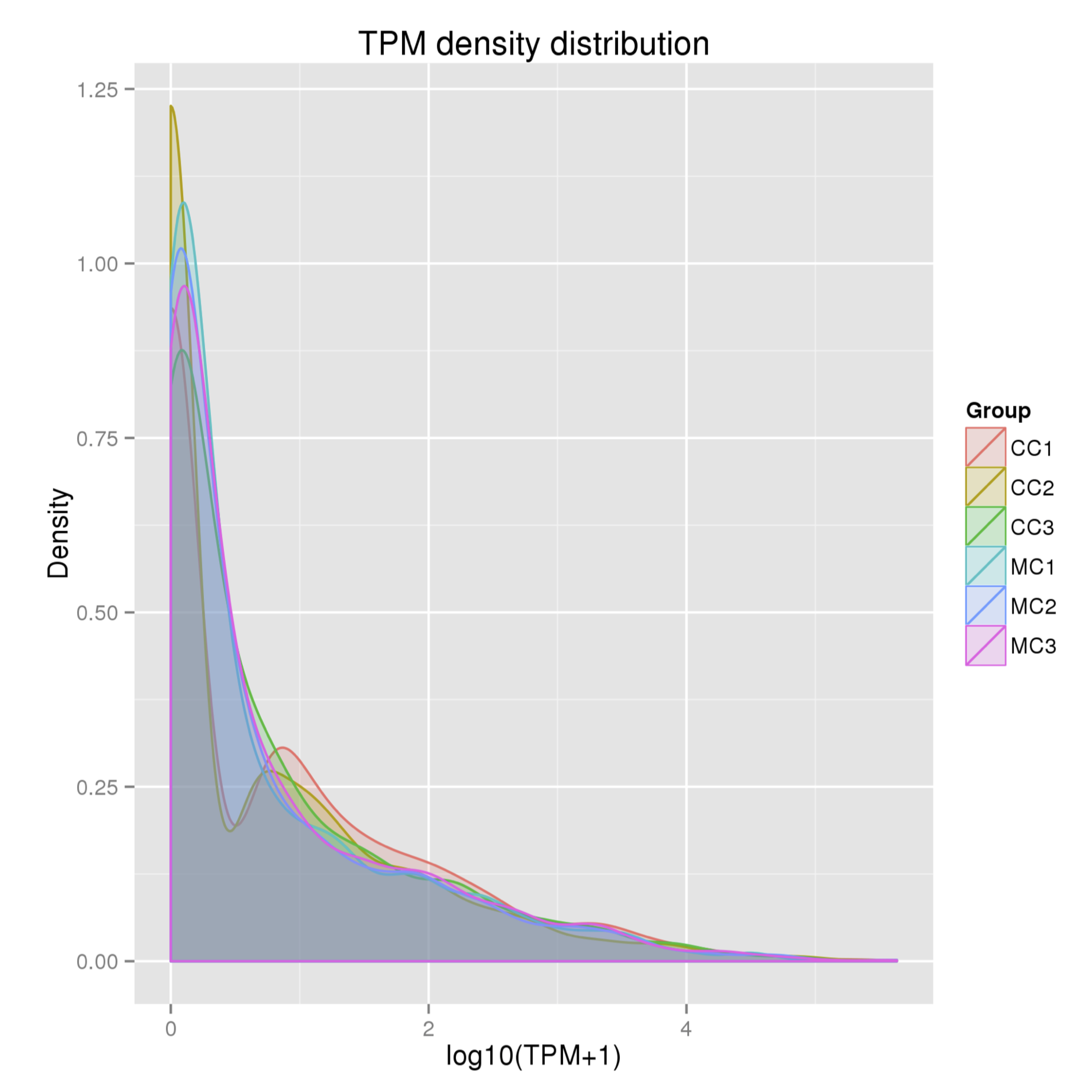


**Figure. S2**. **The density distribution of transcript per million (TPM)**. Horizontal axis: Value of miRNA’s log10 (TPM+1). Vertical axis: the density of matched log10 (TPM+1).


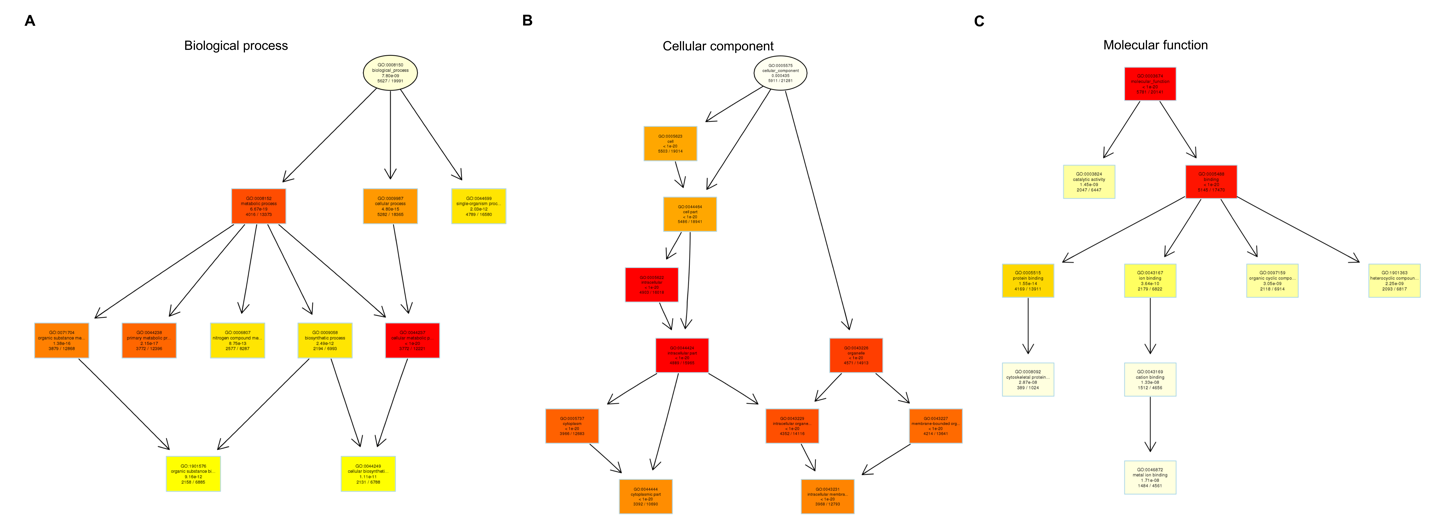


**Figure. S3**. GO function classification target genes of known differential expressed miRNAs in K562 cells after exposure to CAP treatment. From left to right they are biological process (A), cellular component (B), and molecular function (C).
